# Biological mechanisms and applied prospects of mesenchymal stem cells in premature ovarian failure

**DOI:** 10.1097/MD.0000000000030013

**Published:** 2022-08-12

**Authors:** Lan Shi, Zhifen Zhang, Miao Deng, Fangyuan Zheng, Wenhua Liu, Shujin Ye

**Affiliations:** a The Fourth School of Clinical Medicine, Zhejiang Chinese Medical University, Hangzhou, Zhejiang Province, People’s Republic of China; b Department of Obstetrics and Gynecology, Hangzhou Women’s Hospital (Hangzhou Maternity and Child Health Care Hospital), Hangzhou, Zhejiang Province, People’s Republic of China.

**Keywords:** infertility, mesenchymal stem cells, premature ovarian failure, primary ovarian insufficiency

## Abstract

Premature ovarian failure (POF), also known as primary ovarian insufficiency (POI), refers to the loss of ovarian function in women after puberty and before the age of 40 characterized by high serum gonadotropins and low estrogen, irregular menstruation, amenorrhea, and decreased fertility. However, the specific pathogenesis of POF is unexplained, and there is no effective therapy for its damaged ovarian tissue structure and reduced reserve function. Mesenchymal stem cells (MSCs), with multidirectional differentiation potential and self-renewal ability, as well as the cytokines and exosomes they secrete, have been studied and tested to play an active therapeutic role in a variety of degenerative pathologies, and MSCs are the most widely used stem cells in regenerative medicine. MSCs can reverse POI and enhance ovarian reserve function through differentiation into granulosa cells (GCs), immune regulation, secretion of cytokines and other nutritional factors, reduction of GCs apoptosis, and promotion of GCs regeneration. Many studies have proved that MSCs may have a restorative effect on the structure and fertility of injured ovarian tissues and turn to be a useful clinical approach to the treatment of patients with POF in recent years. We intend to use MSCs-based therapy to completely reverse POI in the future.

## 1. Introduction

Primary ovarian insufficiency (POI) is a state of irreversible decline in ovarian function, long-term low estrogen levels can also lead to vasomotor symptoms, urogenital symptoms, osteoporosis, bone loss, induced type II diabetes, cardiovascular and cerebrovascular diseases, psychological diseases, and so on.^[1–3]^ There are many causes of POI, and the etiological analysis of POI is of great significance for clinical treatment. Any factor that causes follicular reduction, accelerated follicular atresia, and abnormal egg function can cause POI. The main clinical causes of POI currently include chromosomal and genetic defects, autoimmune diseases, infectious factors, and medical factors. Genetic factors account for about 20% of the causative factors, mainly due to abnormal X chromosome pairing and genetic mutations in POI patients, failing to produce critical proteins for follicular development and hormone production, which eventually leads to impaired ovarian development and folliculogenesis. Iatrogenic factors mainly point to the damage of chemotherapy, radiotherapy, and operation to the ovary. However, about 50% to 90% of patients with POI have an unclear etiology, and this group of patients is called idiopathic POI. At present, an increasing number of scholars now believe that idiopathic POI is related to autoimmunity. Certain autoimmune diseases can cause direct damage to ovarian tissue or the production of autoantibodies against ovarian tissue, leading to POI.^[4]^ In addition, 5% to 30% of patients with POI may have a combination of other endocrine or autoimmune diseases, including hypothyroidism, hypoadrenalism, systemic lupus erythematosus, and rheumatoid arthritis. Alleviating the adverse physical and mental effects of long-term low estrogen levels on POI patients, improving part of the follicular function, and minimizing the risk of complications are the main clinical treatment objectives of POI recently. At present, the main clinical treatment for POI is hormone replacement therapy, which can relieve the symptoms associated with low estrogen and reduce the risk of long-term cardiovascular disease and osteoporosis.^[5,6]^ However, hormone replacement therapy does not reverse ovarian function and has been shown to increase the risk of breast cancer, venous thrombosis, endometrial cancer, and ovarian cancer. Other measures, such as oocyte and embryo cryopreservation with the aid of assisted reproductive technology at an appropriate age, are used but are not optimal, and there is no effective treatment for the restoration of ovarian function in patients with POI, so research and the development of safe and effective treatments for POI that can repair damaged ovarian tissue structures while improving ovarian reserve function and fertility is essential to improve the quality of life and fertility outcomes of patients with premature ovarian failure.

Mesenchymal stem cells (MSCs) are pluripotent stem cells with significant proliferation, repair potential, and the ability to differentiate into multiple cell lines and exist widely in various tissues.^[7–9]^ MSCs are easy to be isolated, cultured, and amplified, and easy to be introduced and expressed by foreign genes. They always maintain the potential of multidirectional differentiation during long-term culture in vitro and have a stable genetic background. Hundreds of clinical trials are currently underway on MSCs for various diseases, demonstrating the tremendous benefits and potential of MSCs.

## 2. Biological characteristics of MSCs

**Figure 1.**
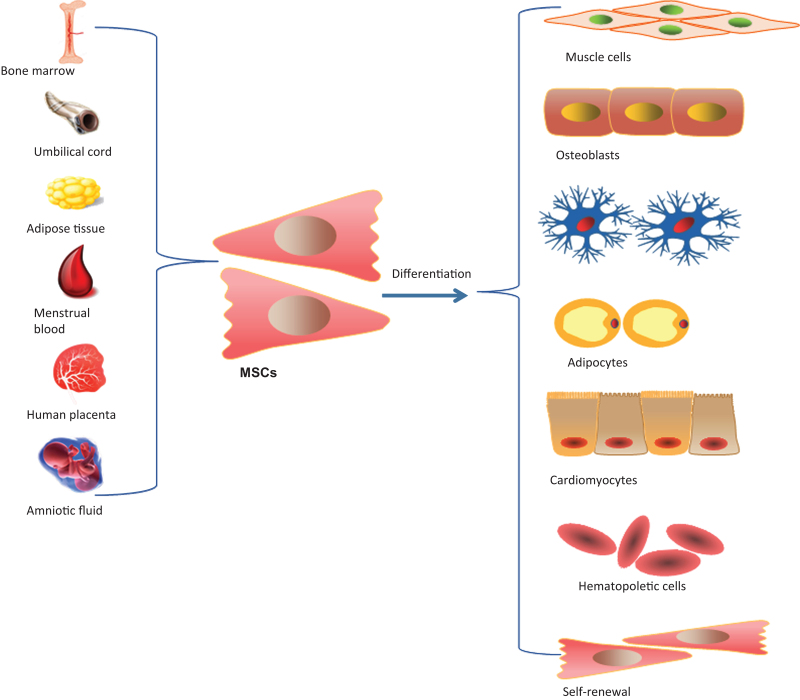
Different sources and differentiation potential of MSCs. This figure shows the multiple sources of MSCs and their potential for self-replication and multidirectional differentiation. MSC = mesenchymal stem cell.

MSCs are the most widely used stem cells in clinical practice, with unique potential for self-renewal and multidifferentiation.^[10]^ The therapeutic potential of MSCs is attributed to their unique biological properties, including differentiation, immunomodulation, and paracrine cytokines. Figure 1 shows the multiple sources of MSCs and their multidirectional differentiation and self-replication potential.

### 2.1. MSCs from different tissue sources

MSCs distribute in connective tissue and organ stroma throughout the body widely. MSCs can be isolated from bone marrow, umbilical cord blood, placenta, amniotic fluid, periosteum, tendon, adipose tissue, menstrual blood, and even brain tissue. It is feasible to extract MSCs from different tissues, and the application of MSCs in the treatment of some immune and ischemic diseases may be promising because of the wide range of sources.^[11]^ Numerous studies have shown that MSCs can differentiate not only into neural cells, osteoblasts, and muscle cells but also into chondrocytes, cardiomyocytes, and islet cells under certain conditions. The multidirectional differentiation potential of MSCs to form specific tissue cells is the theoretical basis for clinical cell replacement therapy. Studies have shown that MSCs not only have the potential of 3-line differentiation in vitro, but also have gene expression profiles, and are the best source of stem cells for tissue engineering and regenerative medicine.^[12]^ After transplantation, MSCs can be induced by certain factors to migrate to the site of damaged tissues and differentiate into specific cell types required for repairing damaged tissues, allowing the local microenvironment of damaged tissues to be reconstructed and participating in the repair of damaged tissues by regulating the immune response, thus MSCs may become a new way to treat POI in clinical practice.

### 2.2. Potential for restoration

MSCs are known to have the potential for self-renewal, differentiation, immunomodulation, and tissue regeneration. MSCs have homing properties at sites of ischemia, inflammation, and other damage, and when the body’s tissues are damaged, MSCs can specifically migrate to sites of inflammation and damage and release multiple cytokines to promote angiogenesis and repair damaged tissues in the local microenvironment and improve the structure and function of damaged tissues.^[11,13,14]^

### 2.3. Potential for differentiation

MSCs have a variety of unique functions, including differentiation potential, colony formation, and self-renewal ability. Previous studies demonstrated that bone marrow MSCs regulated by the Phosphoinositide 3-Kinase/Anaplastic Lymphoma Kinase pathway and can stimulate ovarian microvascular endothelial cell proliferation and increase the expression of angiogenic marker genes in ovarian microvascular endothelial cells through paracrine effects on ovarian endothelial cells, thus promoting ovarian angiogenesis, increasing the density of neovascularization, and effectively restoring ovarian function in POI rat models.^[15]^

## 3. The applications of MSCs in the treatment of POI

In some basic and clinical studies, cell therapy using MSCs is beneficial in alleviating ovarian dysfunction. MSCs of different origins have certain biological characteristics and functional differences, and their mechanisms of action and efficacy in the treatment of POI vary, but the many commonalities of MSCs, such as their multidirectional differentiation potential and stable genetic background, make them ideal candidates for the clinical treatment of POI. To further enhance the therapeutic effect of MSCs on POI, many therapeutic approaches based on MSCs from different sources for premature ovarian failure have been explored and tested. Table 1 summarizes the use of MSCs from different sources in the treatment of POI.

**Table 1:** Studies of mesenchymal stem cells from different sources in POI therapy.

MSCs types	Therapeutic mechanism	References
BMSCs	Decreasing chemotherapy-induced apoptosis and stimulating granulosa cell proliferation	^[16,17]^
	Upregulated the expression of CYP19A1 and StAR genes	^[15]^
	Stimulate angiogenesis in HOVEC cells via the PIK3/ALK pathway	^[18]^
	Genetic and epigenetic regulation of TGF-[scolor_start FADADD]β[/scolor] and Wnt/[scolor_start FADADD]β[/scolor]-catenin and Hippo pathways	
UCMSCs	Increasing the level of free amino acids activates the PI3K pathway, improves lipid metabolism, and reduces monosaccharide concentration	^[19]^
	Recover ovarian function by inducing angiogenesis via the PI3K/AKT signaling pathway	^[10]^
	Improve the ovarian function of chemotherapy-induced POI rats and can pass through NGF/TrkA signaling pathway is regulated	^[20]^
hPMSCs	Improved POI mice high gonadotropin and low estrogen levels promote follicular development and inhibit follicles atresia and granulosa cell apoptosis	^[21]^
	It can increase the kinase activity of Akt, activate the PI3K/Akt signaling pathway, reduce the apoptosis of granulosa cells in the ovary of mice, reduce the ratio of Th17/Tc17 and Th17/Treg cells, and improve ovarian function	^[22,23]^
hAMSC	Secretion of a variety of follicular development-related factors, thereby effectively inhibiting granulosa cell apoptosis, by activating the human luteinized granulosa cell TGF-[scolor_start FADADD]β[/scolor]/Smad pathway to restore ovarian function	^[24]^
	By improving the local microenvironment of the ovary to promote follicular development, granulosa cell proliferation, and hormone secretion function	^[25]^
ADSCs	The role of paracrine cytokines improves ovarian function	^[26]^
	The expression of CXCR4 was downregulated and the ovarian function was improved through the change of gene expression	
HuMenSCs	Repair ovarian damage, promote regeneration and improve ovarian function by secreting FGF2	^[27]^
	Promote the repair of ovarian function through paracrine action	
AFSCs	The exosomes of AFSCs are rich in miR-146a and miR-10a can downregulate the expression of pro-apoptotic proteins and the expression of Caspase-9, thus inhibiting granulosa cell apoptosis and preventing follicular atresia, restoring the function of ovarian injury	^[28,29]^

### 3.1. Pretreatments enhance the therapeutic effects of MSCs in POI

MSCs may have a role in the treatment of many diseases, but their therapeutic efficacy depends on proper and effective transplantation and survival. Researchers have also done a lot of research on how to improve the survival rates of MSCs.

Hypoxic pretreatment has been reported for the survival of MSCs in vivo life has a positive effect.^[30]^ Liu et al^[31]^ showed that after transplantation of hypoxic pretreated bone MSCs (BMSCs) into rats, the rates of vascular remodeling were significantly accelerated, which played a positive role in promoting endothelial cell proliferation and inhibiting endothelial apoptosis. These results indicate that hypoxia preconditioning plays an important role in maintaining MSCs pluripotency, promoting normal embryo development, and mobilizing MSCs homing. Qiao et al^[32]^ reported that heat shock protein can activate autophagy of BMSCs, prevent apoptosis of BMSCs and promote its application in regenerative medicine cell repair therapy.

### 3.2. Gene modification enhances the therapeutic effects of MSCs in POI

A range of genes and microRNAs with defined biological functions have been introduced into MSCs through viral or nonviral vectors to improve their differentiation, immune regulation, homing ability, and increase the chances for success of MSCs therapy in tissue-repair applications.^[33,34]^ MicroRNA-21 is among the earliest discovered miRNAs in mammals, which has been shown to play essential roles in the cellular and physiological pathways in the reproductive system via regulating target genes and is involved in ovarian reserve.^[35,36]^ Li et al^[35]^ showed that MicroRNA-21 may facilitate the development of autoimmune POI via downregulating Pellino-1. Research reported that miR-21 is involved in apoptotic regulation in many cells, miR-21 is also regulatory for the apoptosis of granulosa cells (GCs) and follicular development.^[35–37]^ The research by Fu et al^[36]^ reported that miR-21 can further improve the therapeutic effect of MSCs on POI through decreasing the apoptosis of GCs in MSCs.

Growth differentiation factor 11 is a member of the transforming growth factor-β (TGF-β) superfamily, which has been revealed to play critical roles in inducing endothelial cells proliferation and migration.^[38]^ A recent study confirmed that growth differentiation factor 11 significantly enhanced the potential of MSCs for endothelial differentiation into endothelia-like cells and increased their viability and argued the therapeutic efficacy of MSCs to promote angiogenesis via activation of TGF-β receptor and its downstream Extracellular Signal-Regulated Kinase/eukaryotic initiation factor 4E pathway.^[39]^

### 3.3. MSCs-derived ovarian regenerative patches have positive therapeutic potential

MSCs therapy demonstrated significant potential and availability for regenerative treatment of POI. Many studies have shown that transplanted MSCs can effectively transfer to the target site, carry bioactive molecules to the target site, and exert their therapeutic effects through direct differentiation and paracrine effects.^[40,41]^ However, there are some shortcomings in the clinical application of MSCs therapy, such as the methods of administration, efficiency, immunogenicity, and risk of tumorigenicity. MSCs-derived ovarian regenerative patches (ORP) have been successfully applied to the repair of damaged ovarian. In a study by Zhang et al,^[42]^ MSCs-derived ORP have great potential, they designed an ORP by embedding synthetic MSCs into a clinically approved surgical pelvic scaffold, and the result revealed that ORP can significantly ameliorate ovarian function by the stimulated proliferation of ovarian somatic and inhibited apoptosis under injury stress.

### 3.4. The application of MSCs-derived extracellular vesicles in POI

Extracellular vesicles (EVs) can be readily secreted from MSCs of various origins and fully maintain biologically viability, which can be transferred to damaged cells and perform many beneficial effects such as promoting angiogenesis, immune regulation, anti-fibrosis, and anti-oxidative stress.^[43,44]^ Many research has consistently indicated that MSC-derived EVs transfer proteins, lipids, cytokines, and microRNA between different cell types participate in important biological functions, MSC-derived EVs not only possess the same therapeutic potential as MSCs but also exhibit higher biology stability and lower immunogenicity.^[44,45]^

Yang et al^[10]^ investigated the role of Human Umbilical Cord MSCs-EVs (HUCMSC-EVs) in the POI mice model, they pointed out that inducing angiogenesis and activating the Phosphoinositide 3-Kinase/protein kinase B signaling pathway in ovaries could prevent follicle atresia, and ovarian damage of cyclophosphamide/busulfex-induced POI mice is improved, which may introduce a potential therapeutic for the patients with POI. Similarly, studies by Zhang et al^[46]^ showed that HUCMSC-EVs have the potential of antioxidant and ovarian protection and can reduce cisplatin-induced ovarian damage by playing important roles in resistance to GCs apoptosis oxidative stress and restoring synthesis and secretion of steroid hormone. Moreover, Liu et al^[47]^ demonstrated that HUCMSC-EVs can not only significantly restore the ovarian function of POI mice by overall improving hormone levels and the number of ovarian follicles but also recover their fertility by increasing the number of oocytes retrieved, shortening the time to get pregnant, and increasing the number of offspring.

Therefore, for POI and other female reproductive diseases, MSCs-EVs may be a favorable alternative to MSCs treatment. It should be noticed that although preclinical studies result strongly indicated that the future of MSCs-EVs therapeutics has great value, there is still a lot of challenges imperative to be overcome before putting MSCs-EVs into clinical application. MSCs-EVs will not fully approach its potential in new cell-free therapy until the key issues such as the large-scale production methods, accurate quantification, characterization, and safety profile assessments are solved.^[47,48]^

### 3.5. Tissue engineering of MSCs for POI treatment with excellent therapeutic potential

Tissue engineering is an emerging field that uses the combination of biology cells, engineering methods, and material science to develop or substitute biological functions to recover injured or diseased tissues or organs in vitro or in vivo.^[49]^ The 3-dimensional (3D) cell culture can increase MSCs attachment, survival, proliferation, and therapeutic potentials.^[50]^ Recent studies showed that the 3D culture greatly enhances the therapeutic efficacy of MSCs that are transplanted in injured ovaries by improving their paracrine secretions, engraftment, and survival rates.^[51–53]^

Collagen scaffold robustly increases the therapeutic potential of MSCs by reinforcing their attachment, growth, and migration. First, the collagen/MSCs delivery system repaired ovarian function in POI mice by significantly promoting GCs proliferation, microvasculature regeneration, ovarian angiogenesis, and follicles development.^[54]^ Second, the collagen scaffold transplantation potentially promotes the pregnancy rates and embryos implantation at the injured site of the POI mice via improving estrus cycles, estradiol levels, and follicle development.^[55]^ Last but least, the collagen/MSCs system supported the regeneration of endometrium by locating target site and secreting growth factors, the findings suggested that the collagen scaffold may be helpful for women with thin endometrium who have fertility demands.^[56]^

Therefore, a combination of tissue engineering material and MSCs for transplantation seems to be a beneficial therapeutic option for POI patients.^[49]^ Tissue engineering of MSCs shows effective treatment prospects in the field of regeneration, however, further research should discover the inherent mechanism, and additional investigations are needed to settle the clinical application issues.

## 4. Mechanisms of MSC-based treatment for POI

**Figure 2.**
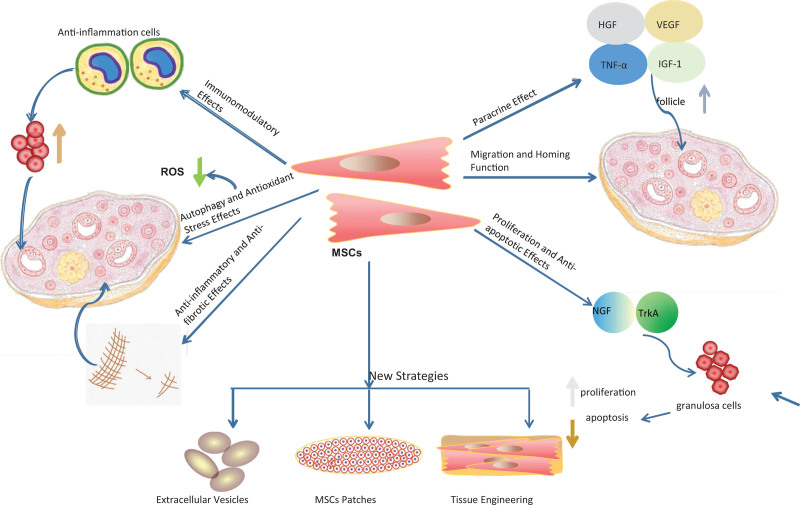
The potential mechanisms and novel strategies of MSCs in POI therapy. Potential therapeutic mechanisms of mesenchymal stem cell therapy include migration and homing function, proliferation, anti-inflammatory, anti-apoptotic, paracrine, immune regulation, anti-fibrosis, and regulation of autophagy and oxidative stress. HGF = hepatocyte growth factor, IGF-1 = insulin-like growth factor 1, MSC = mesenchymal stem cell, NGF = nerve growth factor, TNF-α = tumor necrosis factor-α, TrKA = tyrosine receptor kinase A, POI = primary ovarian insufficiency, VEGF = vascular endothelial growth factor.

MSCs have been extensively investigated as a potential cell-based therapy to rehabilitate ovarian function for POI patients. Transplanting MSCs is a promising therapeutic approach that can promote ovarian regeneration and restore ovarian function through the migration and homing of MSCs into ovarian, paracrine mechanisms, differentiation, proliferation, anti-apoptotic effects, autophagy, oxidative stress regulation, anti-fibrotic effects, and immunoregulation. The potential mechanisms of MSC-based therapy in POI were shown in Figure 2.

### 4.1. Migration and homing function of MSCs to target tissue

The homing function of MSCs can be thought of as transport by endothelium after the arrest of MSCs in tissue vessels.^[13,57]^ The critical challenge of MSC-based treatment is the efficient delivery of cells to the target damaged organ. In the previously published article, Liu et al^[58]^ reported that the intravenous injection of BMSCs can home to the injured tissue site, then fully maintain biologically viability settled in the interstitial of the ovary and allows BMSCs to exert their potentially restorative functions in POI rat models.

Based on the significant role of MSCs in the therapeutic of disease, how to improve the efficacy of their homing has attracted more and more attention.^[49]^ Migration, homing, and locating in the target tissue site for a long time are significant for MSCs’ therapeutic efficacy. Song et al^[59]^ showed that the HUCMSCs transplantation has a positive effect on their settled in ovarian and remained to survive for a long period for efficacy repairing injured tissue and restoring fertility and pregnancy rates in the POI rat models. These findings indicated that the MSCs remaining alive longer in the target issue after homing could result in a long-term therapeutic effect. Remarkably, migration and homing functions of MSCs demand mediation of all sorts of factors.^[60]^ Gabr et al^[61]^ observed that the transplanted BM-MSCs migrated to the injured ovarian stroma after being injected into POI rat models. They could improve the expression levels of ovarian tumor necrosis factor-α and insulin-like growth factor 1, then the 2 cytokines might play a role in attracting MSCs into the target tissue to exert restoration effects. MSCs convey a series of cell adhesion molecules and receptors that could induce migration and homing actions of MSCs to injured organs.^[62]^ Therefore, repair injury ovarian can improve migration and homing function by transplanting MSCs to POI model rats to adjust the long-term therapeutic effect, modifying cytokines level, and so on.

### 4.2. Paracrine effect of MSCs

Recent animal experimental research studies explored those paracrine effects of MSCs play a significant role in the treatment of inflammatory and degenerative diseases. Previous studies have shown that MSCs release soluble paracrine factors such as EVs to produce these paracrine effects. As noted above, MSC-EVs play a positive role in restoring injured ovarian, and soluble paracrine factors secreted by MSCs also have significant therapeutic potential.

Zhang et al^[24]^ discovered that chemo-damaged ovarian function was restored following injection of human amniotic MSC to POI rats, due to MSCs’ ability to increase the expression of automated materials handling, mouse vasa homologue, bone morphogenetic protein 15, and HSA2, which have effects including anti-apoptosis, anti-inflammatory properties, regulation of follicle development, angiogenesis promotion, and repair damaged tissues in the injured ovary. Human adipose-derived MSCs can secrete growth factors such as fibroblast growth factor, insulin-like growth factor 1, hepatocyte growth factor, and vascular endothelial growth factor to modify the microenvironment of POI ovaries, induce GCs apoptosis, promote angiogenesis, and regulate follicular development, thus improving ovarian function and increasing fertility rates in rats with POI.^[63]^ TGF-β has been recognized as the main growth factor of promoting ovarian angiogenesis and follicular development, which strongly inhibits follicle apoptosis in POI rats. Elfayomy et al^[64]^ reported that transplanted human umbilical cord blood MSCs into POI rats repaired damaged ovarian by significantly upregulating the expression of TGF-β.

In summary, transplantation of MSCs shows a significant paracrine effect and play an important role in the treatment of POI; thus, it is imperative to improve the efficacy of paracrine. Kusuma et al^[65]^ reported that MSCs paracrine have been deeply influenced by the extracellular environment. Therefore, several experimental types of research have been exploring ways to enhance MSCs paracrine activity, such as 3D culture systems, dual genetic modification of the MSCs, and more.^[66,67]^

### 4.3. Proliferation and anti-apoptotic effects of MSCs

The transplantation of MSCs provides novel strategies for regenerative medicine applications because MSCs exert a promising proliferation and anti-apoptotic potential.^[68]^ A recent study has shown that MSCs restored ovarian functionality by increasing the proliferation of human GCs and stimulating more estrogen production.^[16]^ Zhou et al^[69]^ demonstrated that transplanting MSC/Matrigel into the ovaries of POI mice significantly improved GCs proliferation rates and functions, follicles number, and ovarian blood vessels development, their findings suggest that the MSC/Matrigel may be an attractive treatment approach for improving endocrine functions and recovering ovulation, restoring injured structures function of ovaries in POI mice. Recent experimental research has investigated a novel source of MSCs, that injected first-trimester human umbilical cord perivascular cells into an animal model of CTX-treated (cyclophosphamide) can be efficiently inhibited the reduction of their ovarian follicle, prevent the damage of ovarian and significantly increase the regenerative potential and fully maintain their ability to proliferate in vitro.^[70]^

In addition, previous studies have shown that MSCs hold a positive potential for regenerative medicine applications due to their positive ability for anti-apoptotic effects. Previous study indicated that transplantation of human placental MSCs notably prevented the apoptosis of ovarian GCs and improved ovarian reserve capacity in POI mice through inhibition of the excessive activation of estrogen receptor stress-related inositol-requiring enzyme 1α signaling pathway.^[71]^ Similarly, another study found that the expression of NGF and tyrosine receptor kinase in ovarian failure rat models were significantly increased, survival rates of primary follicles were notably improved, proliferation ability of GCs promoted obviously, and apoptosis inhibited of GCs remarkably after transplantation of MSCs.^[20]^

In conclusion, MSCs transplantation can regulate ovarian function by enhancing the proliferative effect of POI mice, protecting the proliferation of oocytes and GCs, and reducing the level of ovarian apoptosis. This implies that MSCs transplantation could provide potential and active therapeutic strategy for the clinical treatment of POI patients. Therefore, it is critical to investigate how to improve and provide more efficacious MSCs-related treatment in regenerative medicine applications.

### 4.4. Immunomodulatory effects of MSCs

In MSCs-related therapy, transplantation of MSCs into target tissues has emerged as a promising therapeutic approach because of their immunomodulatory potential. Previous studies have indicated that MSCs exert immunomodulatory activities mainly through cell-cell contact factors.^[72]^ Yin et al^[73]^ have shown that transplantation of human placental MSCs plays a key role to regulate the Treg cells by interacting with various associate cytokines, has a significant effect to recover injured ovarian function in POI animal models. Vasandan et al^[74]^ reported that macrophages play a positive role in the relation of the innate and adaptive immune system, it has been evidenced that MSCs secret prostaglandin E2 to manipulate the metabolism and plasticity of macrophages.

In addition, HUMSCs transplanted into POI mice significantly improved the number of healthy follicles, the expression of the *HOXA10* gene, the function of damaged ovaries, and the level of atretic follicles, T-helper 1/T-helper 2 cytokine ratio, and the number of natural killer cells. These results indicate that HUMSCs treatment is beneficial to regulate the immune system.^[75]^ Several studies investigated that paracrine factors secreted from MSCs are involved in the immunomodulatory effects by interacting with different immune cells, Hu et al^[76]^ indicated that more significant effective therapeutic potential may result from the various cells including MSCs close interactions with natural killer cells.

There is growing evidence that due to the immune modulation effect of MSCs, the transplantation of MSCs can provide a prospective cell-based treatment for POI patients. Although present studies have been exploring various immunomodulatory effects of MSCs, further efforts should be made to identify the biological roles of MSCs in immunological modulation to make MSC transplantation more effective in some clinical applications.

### 4.5. Autophagy and antioxidant stress effects of MSCs

In mammals, autophagy plays a key role in the process of lysosomal degradation by eliminating injured organelles and debris to achieve intracellular material recycling and maintain homeostasis of the internal cellular and tissue environment.^[77–79]^ Autophagy affects the properties of MSCs and may influence their regenerative and therapeutic potential, and previous studies have identified the regulatory role of autophagy and oxidative stress as important mechanisms in the treatment of POI by MSCs.^[49]^

Regulation of autophagy can regulate the characteristics of MSCs and their ability to resist oxidative stress, apoptosis and promote survival. Increased tissue peroxidation and reactive oxygen species can lead to tissue and organ damage and apoptosis, there is growing evidence that various stimuli may regulate apoptosis of antral follicles and GCs of antral follicles by inducing reactive oxygen species, ultimately leading to ovarian dysfunction.^[80]^ Previous studies have shown that autophagy inhibition results in enhanced antioxidant stress effect of MSCs. Young et al^[81]^ found that theca-interstitial cells provided nutrients and hormones to GCs while secreting growth factors are protective against apoptosis of GCs and are essential for normal follicular development. Lu et al^[82]^ indicated that transplantation of HUCMSCs into the POI rat models could restore the reserve function of the damaged ovary by regulating the adenosine monophosphate-activated protein kinase/mammalian target of rapamycin signaling pathway to resist oxidative stress, reducing autophagy, and decreasing apoptosis of theca-interstitial cells. Similarly, Seok et al^[83]^ found that transplanted placenta-derived MSCs were able to migrate to the periphery of follicles in the damaged ovary, inhibiting oxidative stress and reducing GCs apoptosis in the injured ovary by modulating the heme oxygenase-1/heme oxygenase-2 expression in the ovary. The present study indicated that HUCMSCs exerted antioxidant effects by increasing glutathione levels and promoting activation of kinase extracellular regulated protein kinases1/2 pathway to improve cell proliferation and restore ovary function in POI models.^[84]^

Therefore, modulating autophagy in MSCs could improve their resistance to oxidative stress and thus could restore damaged tissues may be exploited as a new therapeutic strategy for regenerative medicine.

### 4.6. Anti-inflammatory and anti-fibrotic effects of MSCs

POI may be associated with ovarian fibrosis, vascular injury, and inflammatory response, and previous studies suggested that MSCs may repair impaired ovarian function through anti-inflammatory and anti-fibrotic mechanisms.^[85]^ Cui et al^[86]^ found that HUMSCs transplanted into the damaged ovaries of POI rat models exerted anti-fibrotic effects through regulating the TGF-β1/Smad-3 signaling pathway, resulting in significant improvement of ovarian function in POI rats. In addition, a previous study reported that human amniotic epithelial cells transplantation effectively inhibited GCs apoptosis while suppressing the inflammatory response to chemotherapy-induced POI, improved follicles quantity, and quality, and reversed ovarian function and fertility in mice with chemotherapy-induced POI.^[87]^ Lai et al^[85]^ have demonstrated that the expression of pro-inflammatory factors tumor necrosis factor-α, TGF-β, interleukin (IL)-8, IL-6, IL-1b, and interferon-γ was significantly reduced after MSCs were injected into POI mice, the results suggest that MSCs may regulate the inflammatory response in damaged ovaries by reducing the activity of inflammatory factors, thereby improving POI and rescuing fertility in infertile mice. Therefore, further studies should explore details on the anti-inflammatory and anti-fibrotic effects of MSCs in improving the quality of life in POI patients.

### 4.7. Differentiation and tissue restoration of MSCs

MSCs are a heterogeneous subpopulation of stromal regenerative cells, a class of stem cells with multidirectional differentiation potential, with the potential to differentiate into germ cells and gametes. Previous studies have shown that human endometrial have pluripotent differentiation properties, and when injected into POI mice can restore the damaged ovarian estrous cycle, acting as tissue repair and regeneration and restoring fertility in POI mice.^[88]^ Similarly, Noory et al^[89]^ found that after human menstrual blood stem cells injected into POI mice could be delivered to injured ovarian tissue and differentiated into GCs, further protecting the function of damaged ovaries by increasing ovarian weight and follicle number. In addition, with MSCs injected into POI rabbits, they were able to differentiate into specific ovarian cells that repair damaged ovarian tissue and restore ovarian regeneration by increasing the secretion of E2 and vascular endothelial growth factor and downregulating the level of follicle-stimulating hormone.^[90]^

Numerous experimental studies have shown that MSCs can localize to the damaged ovarian stroma, differentiate into follicular GCs, promote folliculogenesis and hormone secretion, and play a positive role in the proliferation, survival, and tissue restoration of injured ovarian.^[27,56,91,92]^ Therefore, differentiation and tissue restoration of MSCs may be a new strategy to restore POI patients, however, the mechanism needs to be further studied.

## 5. Clinical practice of MSCs in POI

Based on the significant and positive effect role of MSCs in the treatment of POI animal models and some preclinical studies, there is a growing clinical trial have begun to evaluate the efficacy and safety on MSCs in the treatment of patients with POI.

By 2020, more than 300 trials applying MSCs for clinical therapeutic applications have been completed.^[93]^ A clinical analysis by Yan et al^[94]^ found that after UCMSCs were isolated and cultured according to good manufacturing practices criteria and transplanted into the ovaries of 61 patients diagnosed with POI under the guidance of vaginal ultrasound, no serious complications or side effects were observed in any of the patients, and surprisingly, 4 of the POI patients who had been amenorrheic for a short period of time (<1 year) were successfully conceived after UCMSCs transplantation and delivered healthy babies, demonstrating that the MSCs transplanted into the patients repaired the damaged ovarian function by promoting follicle development and egg production, thus successfully improved the fertility of POI patients. Many novel therapeutic approaches have been investigated in recent years regarding the use of MSCs in regenerative medicine and tissue repair. Previous study has compared the therapeutic effects of transplanting UCMSCs and collagen/UCMSCs into 2 groups of women with POI, respectively, and observed that 1 woman with POI in both groups achieved a normal pregnancy and delivered a healthy baby, these experimental results provide strong evidence that MSCs can be used in the clinical treatment of POI patients to improve ovarian reserve function and enhance fertility.^[95,96]^

Optimizing the transplantation protocol of MSCs improves their safety and efficacy in clinical practice, so exploring the appropriate methods for the clinical application of MSCs is fundamental to effective cell-based therapy. Several studies have been carried out to address the issues that different sources of MSCs, methods of culture and preservation, pretreatment, mode of administration and site of administration may affect their properties, function and safety and therapeutic efficacy after transplantation.^[11,49]^

Numerous studies have shown that the survival rates and therapeutic potential of MSCs in injured tissues can be improved by genetic modification, hypoxia preconditioning, heat shock preconditioning, and the combination of MSCs with biomaterials.^[30–32,51–56,96]^ Currently, MSCs administered by local injection, intravenous injection, and arterial injection, of which local injection is the most common, but local injection can lead to uneven dispersion of MSCs, edema at the lesion site, and the need for multiple injections.^[97]^ Previous study has demonstrated that intravenous injection of MSCs is less invasive and that MSCs transplanted intravenously can cross the capillary barrier to reach the target tissue directly since MSCs are at a certain distance from the target tissue, the number of MSCs reaching the target site and their efficacy may be affected.^[98]^ Thus, the arterial injection may be a promising mode of drug delivery, however, the arterial injection has the potential to cause damage to the microvasculature, producing an inflammatory response and the formation of thrombi that can lead to organ failure. The optimized protocol of MSCs will be gradually applied to the clinical treatment of POI after standardizing the preparation and ensuring the safety of application. With further comprehensive and larger clinical practice, the optimized MSCs protocol will be gradually applied to the clinical treatment of POI and other regenerative medicine after standardizing the preparation and ensuring the safety of application.

## 6. Conclusion

Premature ovarian insufficiency (POI) is a complex and serious endocrine disease that severely affects the quality of life and fertility of females.

In recent years, many experimental studies and clinical trials provided preliminary evidence that MSCs have a good therapeutic effect in repairing ovarian function and restoring reproductive functions of patients with POI, which has brought hope for POI females. New therapeutic strategies such as MSCs-derived ORP, MSCs-derived EVs, gene-modified MSCs, and collagen/MSCs have significantly increased the efficacy of MSCs in the treatment of POI. Although several new MSCs-derived products have been approved for marketing in some countries, further comprehensive and larger-scale experimental studies are needed to fully assess the efficacy and safety of their clinical therapeutic applications. At present, there is no standardized treatment plan for the clinical application of MSCs therapy, and further research is needed to explore the best source, route, site, timing, dose, and frequency of MSCs therapy.

Therefore, MSCs-based POI therapy is difficult to use in clinical practice in the short term due to uncertainty about optimal protocols and ethical issues. Previous laboratory-based studies and preclinical or clinical practices have found that MSCs transplantation treatment is an effective option for the therapeutic of POI due to the multidirectional differentiation, wide availability, and accessibility of MSCs. The therapeutic potential of MSCs is due to their multiple biological properties. Gene-modified MSCs, pretreated MSCs, and cell-free therapy of MSCs are new strategies for the clinical application of MSCs, which can improve the efficacy and safety of POI patients and avoid side effects of drugs.

## Acknowledgments

We would like to express our gratitude to our all team members for their assistance and constant support provided by them.

## Author contributions

Conceptualization: Zhifen Zhang, Lan Shi.

Supervision: Lan Shi, Miao Deng, Fangyuan Zheng.

Visualization: Shujin Ye, Wenhua Liu.

Writing – original draft: Lan Shi.

Writing – review & editing: Zhifen Zhang, Wenhua Liu.
